# Supplementary figures and images for: Foveavelia, a new South American genus of Veliinae (Hemiptera: Heteroptera: Veliidae)
Source: PeerJ. 2024 Mar 21;12:e16772. doi: 10.7717/peerj.16772 (PMC10961057; doi:10.7717/peerj.16772)

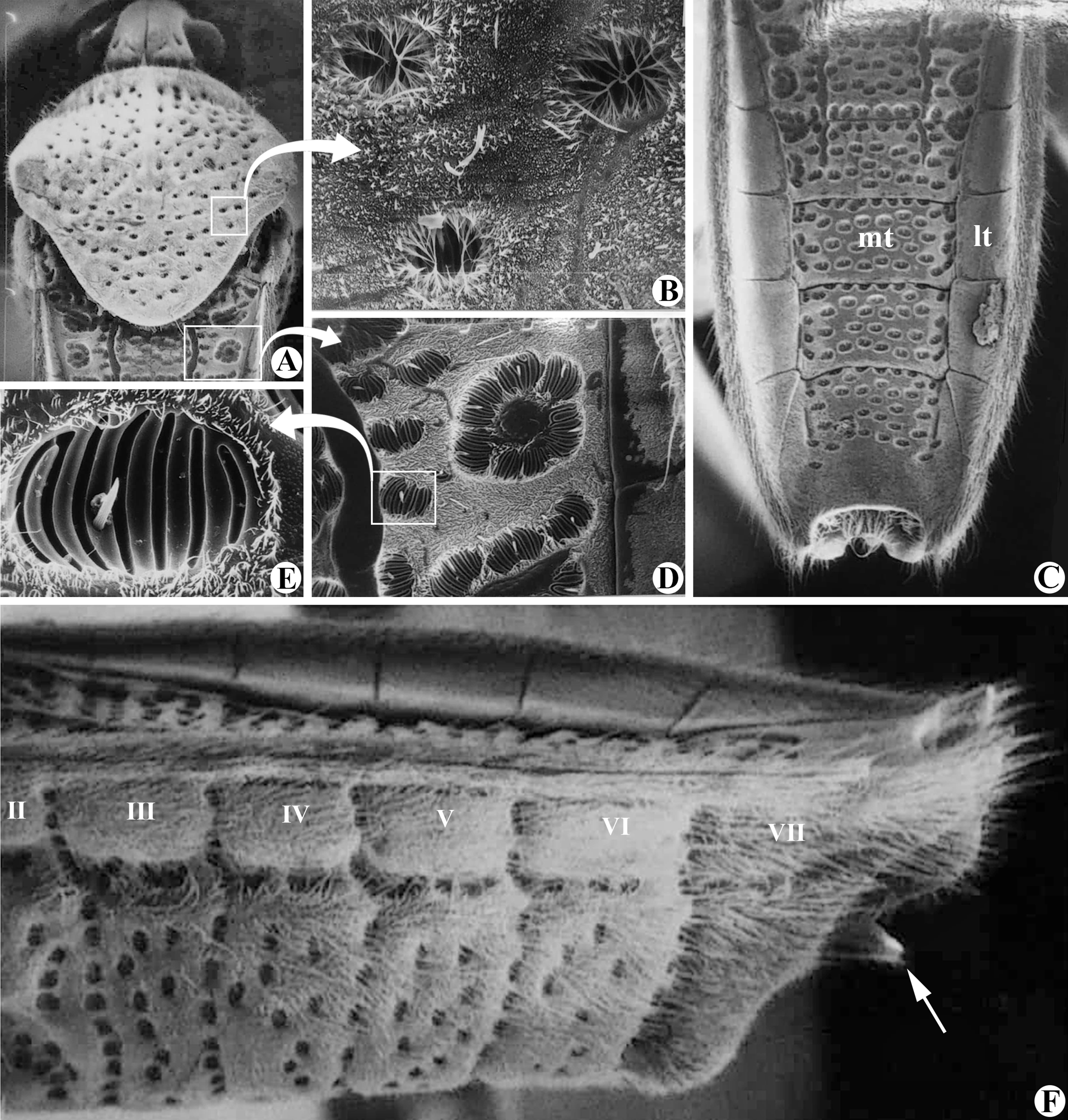

Supplement: Supplemental Information 2 — (A) Dorsal view of head, pronotum and part of abdomen (wings removed). (B) Pronotal punctures. (C) Male abdomen in dorsal view (wings and genital capsule removed). (D) Part of abdominal mediotergite II with suboval punctures. (E) Suboval puncture of abdominal mediotergite IV in detail. (F) Male abdomen in lateral view (wings and genital capsule removed), white arrow indicates posteroventral margin of abdominal segment VIII acuminating distally. mt = mediotergite, lt = laterotergite. Photographs provided by Dr. Silvia Mazzucconi. [file peerj-12-16772-s002.png]

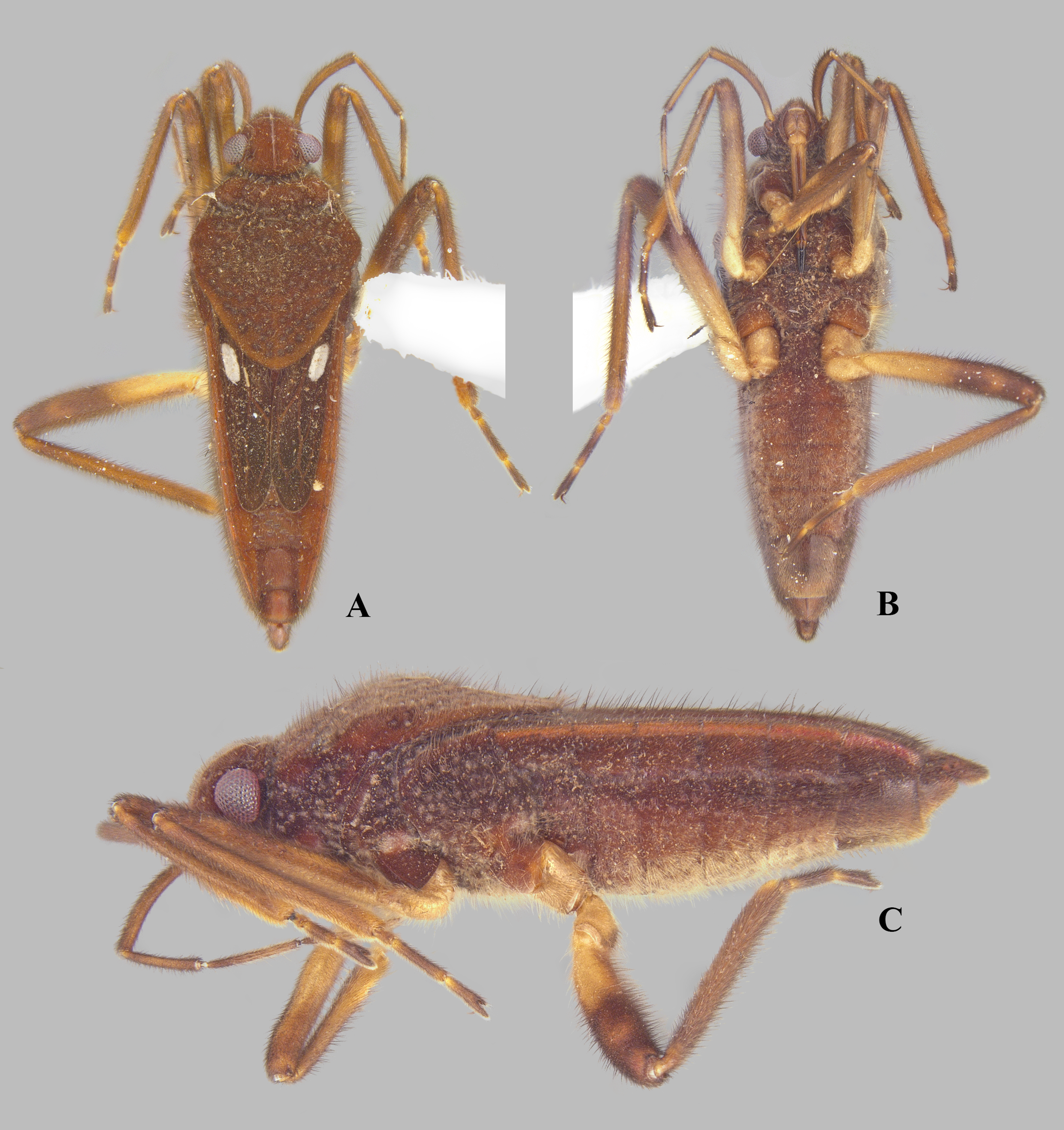

Supplement: Supplemental Information 5 — (A) Dorsal, (B) ventral, and (C) lateral habitus. [file peerj-12-16772-s005.png]

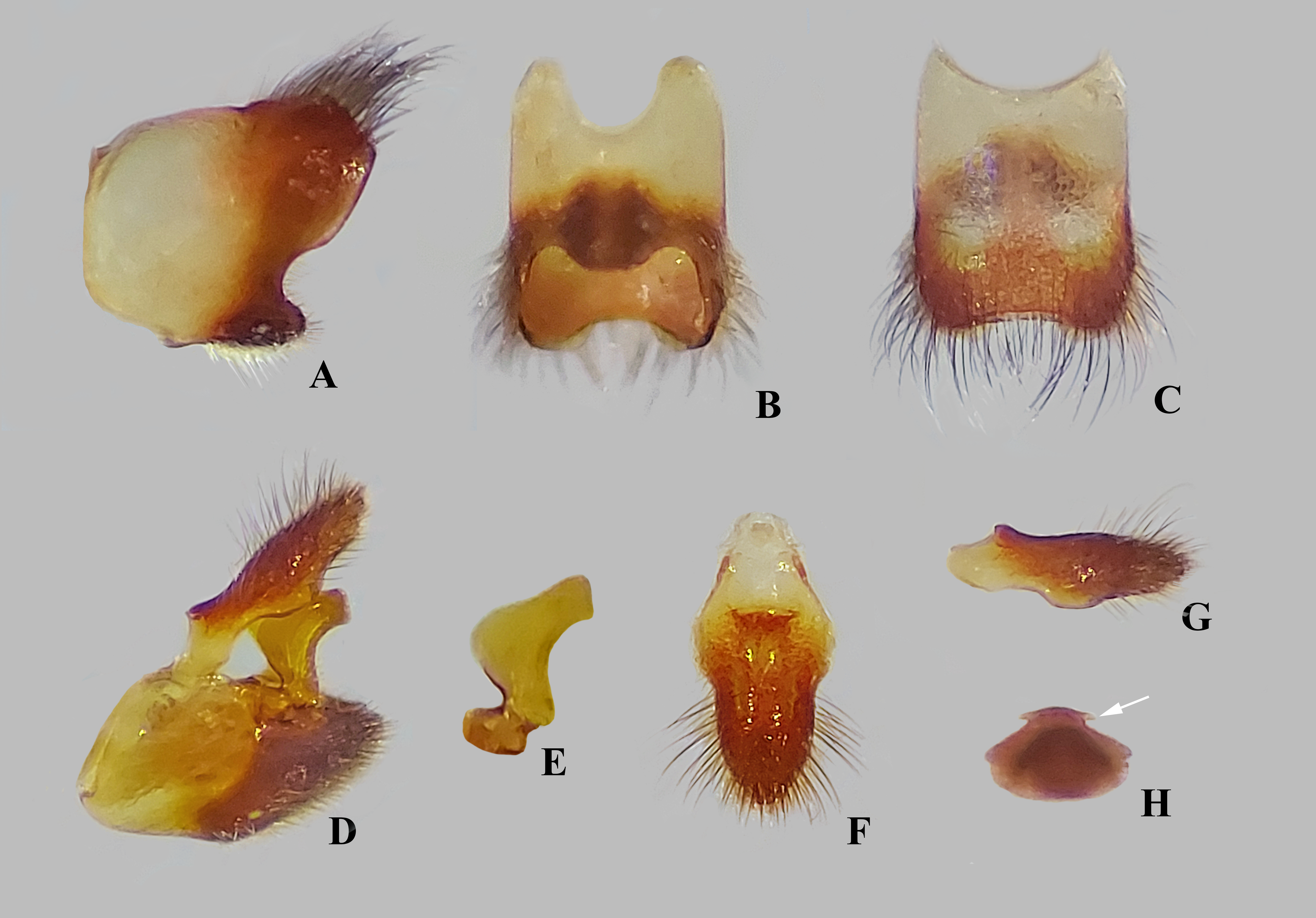

Supplement: Supplemental Information 6 — (A–C) Abdominal segment VIII, (A) lateral, (B) ventral, and (C) dorsal views. (D) Genital capsule in lateral view. (E) Left paramere in lateral view. (F–H) proctiger, (F) dorsal, (G) lateral, and (H) frontal views, white arrow indicates pair of anterodorsal projections. [file peerj-12-16772-s006.png]
